# A Novel Thermostable GH3 *β*-Glucosidase from* Talaromyce leycettanus* with Broad Substrate Specificity and Significant Soybean Isoflavone Glycosides-Hydrolyzing Capability

**DOI:** 10.1155/2018/4794690

**Published:** 2018-10-23

**Authors:** Xinxin Li, Wei Xia, Yingguo Bai, Rui Ma, Hong Yang, Huiying Luo, Pengjun Shi

**Affiliations:** Key Laboratory for Feed Biotechnology of the Ministry of Agriculture, Feed Research Institute, Chinese Academy of Agricultural Sciences, Beijing 100081, China

## Abstract

A novel *β*-glucosidase gene (*Bgl3B*) of glycoside hydrolase (GH) family 3 was cloned from the thermophilic fungus* Talaromyce leycettanus* JM12802 and successfully expressed in* Pichia pastoris*. The deduced Bgl3B contains 860 amino acid residues with a calculated molecular mass of 91.2 kDa. The purified recombinant Bgl3B exhibited maximum activities at pH 4.5 and 65°C and remained stable at temperatures up to 60°C and pH 3.0−9.0, respectively. The enzyme exhibited broad substrate specificities, showing *β*-glucosidase, glucanase, cellobiase, xylanase, and isoflavone glycoside hydrolase activities, and its activities were stimulated by short-chain alcohols. The catalytic efficiencies of Bgl3B were 693 and 104/mM/s towards* p*NPG and cellobiose, respectively. Moreover, Bgl3B was highly effective in converting isoflavone glycosides to aglycones at 37°C within 10 min, with the hydrolysis rates of 95.1%, 76.0%, and 75.3% for daidzin, genistin, and glycitin, respectively. These superior properties make Bgl3B potential for applications in the food, animal feed, and biofuel industries.

## 1. Introduction

Plant cell walls contain three major polymers, i.e., cellulose, hemicellulose, and lignin [1]. Cellulose is the largest component and consists of glucose residues linked by a *β*-1,4-glycosidic bond [2, 3]. It can be degraded into glucose through the cooperation of three types of enzymes, that is, endo-*β*-1,4-glucanase (EC3.2.1.4), exoglucanase (also known as cellobiohydrolase, EC3.2.1.91), and *β*-glucosidase (EC3.2.1.21) [4, 5].

*β*-Glucosidase, also referred to as *β*-d-glucoside glucohydrolase, is a key rate-limiting enzyme that is capable of specifically catalyzing the hydrolysis of *β*-1,4-glycosidic linkages that existed in oligosaccharides, alkyl- or aryl *β*-d-glucosides, and cyanogenic glycosides from the nonreducing ends [6]. The classification of *β*-glucosidase generally follows the CAZY dataset (http://www.cazy.org), in which glycoside hydrolases (GH) are divided into 153 families. The enzymes having *β*-glucosidase activities are confined in the families GH1, 3, 5, 9, 30, and 116 [7], and those of GH3 contain three domains: the (*β*/*α*)_8_ bucket domain, the (*β*/*α*)_6_ sandwich domain, and the Fn3-like region [8]. Glucosidases are widely used in many industries. In the flavor field, *β*-glucosidase can catalyze the production of flavor compounds and remarkably improve food aroma [9, 10]. In the animal feed, *β*-glucosidase is supplemented to convert soybean isoflavone glycosides [5, 11]. *β*-Glucosidase is also used in the detergent, cosmetic, and food products [12].

The thermophilic fungus* Talaromyces *is well-known for producing various extracellular enzymes [13, 14]. Considering the great potential of thermophilic *β*-glucosidases for application in the food and feed field, a novel GH3 *β*-glucosidase-encoding gene from the thermophilic* Talaromyces leycettanus* was overexpressed in* Pichia pastoris* in the present study. The enzymatic properties were characterized comprehensively. The recombinant *β*-glucosidase was found to have broad substrate specificity, high catalytic efficiency of fiber oligosaccharides, and great soybean isoflavone glycosides-degrading capability, thus representing a promising enzyme candidate for application in the feed field.

## 2. Experimental Section

### 2.1. Strains, Media, Vectors, and Chemicals

The filamentous fungus* T. leycettanus* JCM12802 was purchased from Japan Collection of Microorganisms and cultured at 45°C in the wheat bran medium [7].* Escherichia coli *Trans1-T1 (TransGen, Beijing, China) and* Pichia pastoris* GS115 (Invitrogen, Carlsbad, CA) used for gene cloning and heterologous expression were cultured at 37°C and 30°C, respectively. Luria-Bertani (LB) medium supplemented with 50 *μ*g/ml ampicillin and yeast peptone dextrose medium were used for the cultivation of prokaryotic and eukaryotic cells, respectively. The vectors pEASY-T3 (TransGen) and pPIC9 (Invitrogen) were applied for cloning and expression, respectively. The DNA purification kit,* LA* Taq DNA polymerase, and restriction enzymes were purchased from TaKaRa (Otsu, Japan). The* FastPfu* DNA polymerase and Fungal DNA Mini kit were purchased from TIANGEN (Beijing, China) and the Omega Biotek (Doraville, GA), respectively. The total RNA isolation system kit and T4 DNA ligase were purchased from Promega (Madison, WI). The restriction endonucleases and endo-*β*-*N*-acetylglucosaminidase H (Endo H) were obtained from New England Biolabs (Ipswich, MA). Substrates 4-nitrophenyl *β*-glucopyranoside (*p*NPG), barley *β*-d-glucan, medium viscosity carboxymethylcellulose sodium (CMC-Na), lichenan, beechwood xylan Avicel PH-101, amygdalin, and six isoflavone standards (daidzin, genistin, glycitin, daidzein, genistein, and glycitein) were supplied by Sigma-Aldrich (St. Louis, MO) [7]. Xyloglucan, laminarin, and oligosaccharides (cellobiose, cellotriose, cellotetraose, cellopentaose, and laminaritetraose) were supplied by Megazyme (Wicklow, Ireland). All other chemicals were of analytic grade and commercially available.

### 2.2. Gene Cloning and Sequence Analysis

The mycelia of strain JCM12802 were collected after 4-day-growth in wheat bran medium. The genomic DNA and total RNA were extracted and purified in accordance with the manufacturer's instructions of Fungal DNA Mini kit and the SV Total RNA Isolation System, respectively. TransScript® One-Step gDNA Removal and cDNA Synthesis SuperMix kit (TransGen) were used for the production of cDNAs.

According to the conserved regions of fungal *β*-glucosidases of GH3, two conserved sequences SSNIDD and GLDMT (A) MPGD (S) were identified, and a degenerate primer set DP-F and DP-R (Table S1) was designed accordingly to amplify the core region of the objective gene. The purified PCR products with target size were linked with the cloning vector pEASY-T3 (TransGen), which was then transformed into* E. coli* Trans1-T1 for cloning and sequencing. According to the sequencing results, two specific primer sets (Table S1) were designed to amplify the up- and down-stream sequences. The PCR products were purified, sequenced, and assembled with the core region to give the full-length *β*-glucosidase gene* Bgl3B*. According to the sequence of* Bgl3B*, two expression primers (Table S1) were designed to amplify the cDNA fragment coding for the mature Bgl3B without the signal peptide-coding sequence. An annealing temperature of 60°C was set.

The nucleotide sequences of* Bgl3B* were assembled by the Vector NTI 10.0 software (Invitrogen). Sequence comparisons with known GH3 *β*-glucosidase sequences were accomplished by using the BlastN, BlastX, and BlastP at NCBI (http://www.ncbi.nlm.nih.gov/BLAST) based on the homology search. Alignment of multiple protein sequences was conducted using the ClustalX software (http://www.clustal.org/) and rendered by the ESPript 3.0 (http://espript.ibcp.fr/ESPript/cgi-bin/ESPript.cgi). The potential signal peptide was predicted to use the SignalP 4.0 server (http://www.cbs.dtu.dk/services/SignalP/). The online programs NetNGlyc 1.0 Server (http://www.cbs.dtu.dk/services/NetNGlyc/) and NetOGlyc 4.0 Server (http://www.cbs.dtu.dk/services/NetOGlyc/) were used to predict the different type glycosylation sites. The three-dimensional structures of Bgl3B and its complex with cellobiose were homology modeled with the *β*-glucosidase,* Afβ*G, from* Aspergillus fumigatus* (PDB: 5FJI_A) by applying the Discovery Studio v2.5 (Accelrys, San Diego, CA).

### 2.3. Expression and Purification of the Heterologous Protein

The cDNA fragment of* Bgl3B *and vector pPIC9 were both digested by* Eco*RI and* Not*I and then ligated to construct the recombinant plasmid pPIC9-*Bgl3B*. The sequence-verified recombinant plasmid pPIC9-*Bgl3B* was then linearized by* Bgl*II and purified with DNA purification kit. The purified products were transformed into* P. pastoris* GS115 competent cells via electroporation using the Gene Pulser X cell Electroporation System (Bio-Rad, Hercules, CA). Positive transformants were screened on minimal dextrose (MD) plates and grown in corresponding medium of different stages according the previous methods [7, 15]. The cell-free culture supernatants were gathered by high-speed centrifugation (12,000* g*) at 4°C for 10 min and concentrated using a Vivaflow ultrafiltration membrane (Vivascience, Hannova, Germany) that can trap protein with a molecular mass of more than 3 kDa. The crude enzyme was further purified using a FPLC HiTrap Q Sepharose XL 6 ml column (GE Healthcare, Uppsala, Sweden) according to the previous methods of protein purification [7, 15]. Fractions showing *β*-glucosidase activities (as described below) were identified using sodium dodecyl sulfate-polyacrylamide gel electrophoresis (SDS-PAGE) and pooled for further studies.

### 2.4. Enzyme Activity Assays

#### 2.4.1. pNP Method

When using* p*NPG as the substrate, the standard systems containing enzyme solution (250 *μ*l) and 1 mM of* p*NPG in 100 mM citric acid-Na_2_HPO_4_ buffer (250 *μ*l, pH 4.5) were treated at 65°C for 10 min and terminated by the addition of 1 M of Na_2_CO_3_ (1.5 mL). After cooling to room temperature, the amount of the liberated* p*-nitrophenol (*p*NP) was measured spectrophotometrically at 405 nm [7, 15]. One unit (U) of *β*-glucosidase activity was defined as the amount of enzyme which generated 1 *μ*mol of* p*NP per min under the assay conditions [15, 16].

#### 2.4.2. GOD-POD Method

To measure the Bgl3B activities towards cellobiose (4 mM), laminaritetraose (5 mM), cellotriose (1%), cellotetraose (1%), cellopentaose (1%), gentiobiose (1%), genistin (1%), daidzin (1%), or amygdalin (1%), the GOD-POD method was used to determine the amount of reducing glucose with a commercial kit (Biosino, Beijing, China) [15]. The standard reaction systems containing enzyme solution (70 *μ*l) and 2 mM cellobiose (70 *μ*l) in 100 mM citric acid-Na_2_HPO_4_ buffer (pH 4.5) were incubated at 65°C for 10 min, terminated in a boiling water bath and added with moderate GOD-POD coloring solution (2.1 ml) [7]. The amount of released glucose was estimated by measuring the absorbance at 520 nm. One unit of *β*-glucosidase activity was defined as the amount of enzyme which generated 1 mmol of glucose per min under the assay conditions [17].

#### 2.4.3. Dinitrosalicylic Acid (DNS) Method

DNS method was used to measure the Bgl3B activity towards polysaccharides [18]. The reaction systems containing substrate solution (900 *μ*l, 1% [w/v] of laminarin, barley *β*-d-glucan, Avicel, xylan, or CMC-Na or 0.5% [w/v] of lichenan in 100 mM citric acid-Na_2_HPO_4_, pH 4.5) and 100 *μ*l of enzyme were treated at 65°C for 10 min. The reactions were stopped by the addition of DNS reagent (1.5 ml), followed by a boiling water bath for 5 min and cooling to room temperature. The absorbance at 540 nm was measured. One unit (U) of enzyme activity was defined as the amount of enzyme which generated 1 *μ*mol of reducing sugar per min under the assay conditions [19]. A Bio-Rad microplate absorbance reader was applied, and all assays were measured three times.

### 2.5. Biochemical Characterization

#### 2.5.1. pH and Temperature Properties

The enzyme properties of purified recombinant Bgl3B were determined by using the* p*NP method. The activity-pH profile was measured at 60°C in 100 mM citric acid-Na_2_HPO_4_ (pH 3.0–8.0) and 100 mM Tris-HCl (pH 8.0–9.0) for 10 min [15]. The activity-temperature profile was measured at the optimal pH and different temperatures (30–90°C) for 10 min. The pH stability and thermal stability were estimated by measuring the residual enzyme activities under optimal conditions (pH 4.5, 65°C, and 10 min) after preincubation of the enzyme in different buffers (pH 2.0–10.0) at 37°C for 1 h or at the optimal pH and 60°C or 70°C for different periods (5, 10, 20, 30, and 60 min) without substrate.

#### 2.5.2. Effect of Chemicals

To estimate the effects of various chemical reagents on the activity of Bgl3B, the standard reaction system supplemented with 5 mM of Pb^2+^, Na^+^, Ni^2+^, K^+^, Cu^2+^, Li^+^, Fe^3+^, Ag^+^, Cr^3+^, Mn^2+^, Zn^2+^, Ca^2+^, Co^2+^, Mg^2+^, SDS, EDTA, or *β*-mercaptoethanol was subject to enzyme activity assay and compared to the blank control without any chemical addition [7, 20].

#### 2.5.3. Effect of Alcohols

Alcohols are strong nucleophile reagents of *β*-glucosidases [21]. The effects of methanol, ethanol, and propanol on Bgl3B activities against* p*NPG were also studied by measuring the residual enzyme activities in the presence of different concentrations of alcohols under optimal reaction conditions (pH 4.5 and 65°C for 10 min). The alcohol stability of Bgl3B was determined by measuring the residual activities after pretreatment with ethanol of different concentration (up to 50%) at pH 4.5 and 30°C for 4 h.

### 2.6. Substrate Specificity and Kinetic Parameters

#### 2.6.1. Substrate Specificity

The substrate specificity of the purified recombinant Bgl3B was determined under optimal reaction conditions using various* p*NP derivatives (1 mM), oligosaccharides (5 mM), polysaccharides (1% [w/v]), or soybean isoflavone glycosides (1% [w/v]) as the substrate.

#### 2.6.2. Kinetic Parameters

The kinetic parameters (*K*_m_,* V*_max_, and* k*_cat_) of Bgl3B were estimated at pH 4.5 and 65°C for 5 min, with different concentrations of cellobiose (1 to 10 mM) or* p*NPG (0.2 to 1.5 mM) as the substrate. The experiments were repeated three times, and each experiment had three replicates. The data were calculated and analyzed according to the Lineweaver-Burk method [7].

### 2.7. Assay of Glucose Tolerance

The inhibitory effect of glucose on the purified recombinant Bgl3B was assayed by fitting the Dixon plot [22]. The enzyme (500 *μ*l) was added to glucose solution of different concentrations (12 ml, 0.01–2.0 M), followed by 1 h-incubation at room temperature. The systems contained* p*NPG (12.5 ml, 3 or 4 mM), 100 mM citric acid-Na_2_HPO_4_ buffer (250 ml, pH 4.5), and the same amount of glucose [15]. The residual enzyme activities were measured under optimal conditions (pH 4.5 and 65°C for 10 min). The* K*i value was determined by drawing two linear functions of reaction velocities and glucose concentrations in the presence of 3 or 4 mM of* p*NPG.

### 2.8. Analysis of the Hydrolysis Products of Soybean Isoflavone Glycosides

A commercial *β*-glucosidase from Sigma-Aldrich (G4511, from almonds) was used for comparison of the soybean isoflavone glycosides-degrading ability with Bgl3B. The systems containing soybean flour solution (50 *μ*l, 10% [w/v]) and each enzyme solution (200 *μ*l, 0.05 U) in citric acid-Na_2_HPO_4_ buffer (100 mM, pH 4.5) were incubated at 37°C for 10 min, and the reactions were terminated in an ice water bath. The hydrolysates were collected by high-speed centrifugation (12,000* g*, 4°C and 10 min) and ultrafiltrated using a Vivaflow ultrafiltration membrane (Vivascience) that can trap a molecular mass of more than 3 kDa and were subject to the high-performance liquid chromatography (HPLC) analysis with the Waters HP1100 (Milford, MA) equipped with a C18 column (5 mm × 250 mm) [15]. The chromatograms were detected at 254 nm. The calibration curves of six isoflavone standards were prepared to calculate the isoflavone contents in samples. The reactions without any enzyme were set as blank controls. Each experiment had three replicates.

## 3. Results

### 3.1. Gene Cloning and Sequence Analysis

The cDNA of* Bgl3B* (GenBank accession number: MF445381) from* T. leycettanus* JCM12802 consists of 2,583 base pairs and encodes 860 amino acid residues with the calculated isoelectric point and molecular weight values of 4.83 and 91.2 kDa, respectively. SignalP analysis indicated that deduced Bgl3B contains of a putative signal peptide of 19 amino acid residues at the N-terminus. The enzyme contains a total of thirty-six potential glycosylation sites (12* N*-glycosylation and 24* O*-glycosylation sites, respectively). Sequence alignment showed that deduced Bgl3B had the highest identity of 78% with a thermostable *β*-glucosidase from* Thermoascus aurantiacus* IFO9748 (GenBank accession no. AAZ95587.1) and 75% identity with the structure-resolved *β*-glucosidase from A*. fumigatus* (PDB no: 5FJI). The results of BLAST analysis and multiple alignments indicated that Bgl3B belongs to the family GH3 (Figure S1). Homology modeling indicated that deduced Bgl3B contains three typical domains of GH3 *β*-glucosidases (Figure S2): the N-terminal TIM-barrel domain, the C-terminal *α*/*β* sandwich domain, and the fibronectin type III (Fn3)-domain [8]. The putative catalytic residues are D261 and E490.

### 3.2. Expression and Purification of the Recombinant Bgl3B

**Figure 1:**
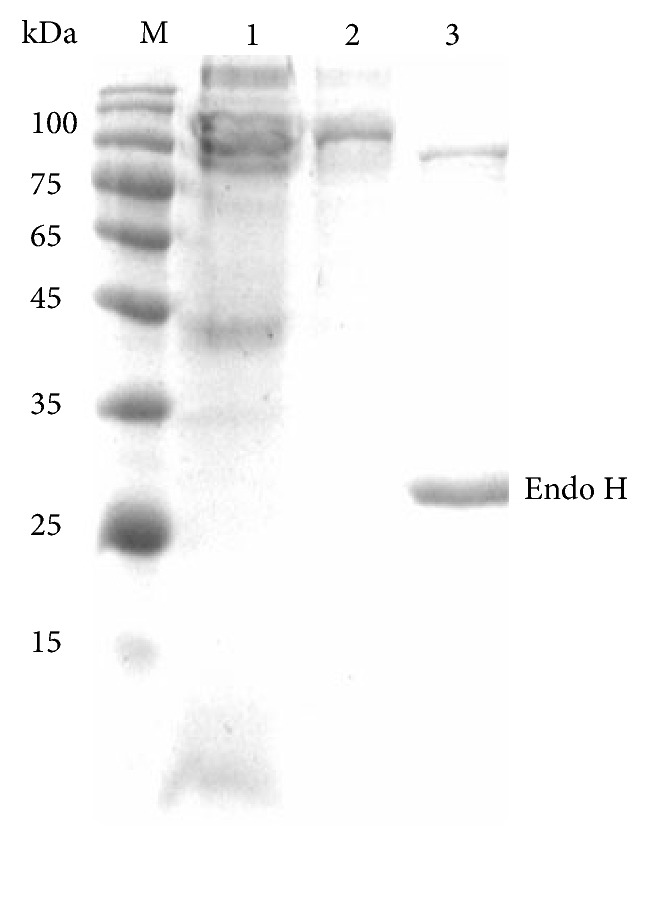
SDS-PAGE analysis of the purified recombinant Bgl3B. Lane M, the standard protein molecular weight markers; lane 1, the crude enzyme; lane 2, the purified enzyme; and lane 3, the purified enzyme after digestion with Endo H.

The cDNA fragment of* Bgl3B* without the signal peptide-encoding sequence was overexpressed in* P. pastoris* GS115. After methanol induction in BMMY medium, the culture supernatants showed significant *β*-glucosidase activities of 1.5 U/ml. This result indicated that the recombinant Bgl3B was successfully expressed and secreted into the medium. The enzyme was purified into electrophoretic homogeneity as shown in SDS-PAGE (Figure 1). The apparent molecular mass of the purified recombinant Bgl3B was close to 100.0 kDa, which is higher than the theoretically predicted molecular mass (91.2 kDa). After 1 h-digestion with Endo H at 37°C, the protein showed a molecular mass of approximately 91.0 kDa. It indicated that* N*-glycosylation occurred in recombinant Bgl3B during the heterologous expression in* P. pastoris*.

### 3.3. Biochemical Properties of the Purified Recombinant Bgl3B

**Figure 2:**
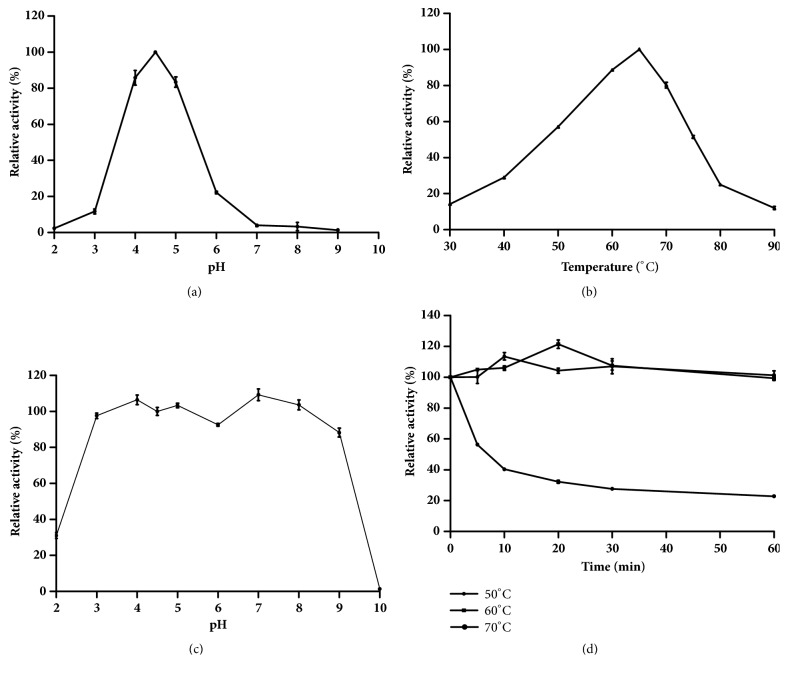
Biochemical characterization of the purified recombinant Bgl3B. (a) Effect of pH on the Bgl3B activity; (b) effect of temperature on the Bgl3B activity; (c) pH stability; (d) thermostability. Each value in the panel represents the mean ± SD (n = 3).

The enzymatic properties of the purified recombinant Bgl3B were determined using* p*NPG as the substrate. When assayed at 60°C, the pH optimum for Bgl3B activity was found to be 4.5 (Figure 2(a)). At pH 4.5, the purified Bgl3B exhibited optimal activity at 65°C, and remained >50% of the maximum activity at 50–75°C (Figure 2(b)). The enzyme showed stability over a wide pH range, retaining >80% of the original activity at pH from 3.0 to 9.0 (Figure 2(c)). And the enzyme was stable at 60°C, but lost >80% of the activity at 70°C after 1 h (Figure 2(d)).

The effects of various chemicals on the activities of purified Bgl3B are shown in Table 1. The majority of chemicals tested had little or no effect on Bgl3B. But when Ag^+^ and Fe^3+^ were added, the enzyme lost more than 90% of the activities.

**Figure 3:**
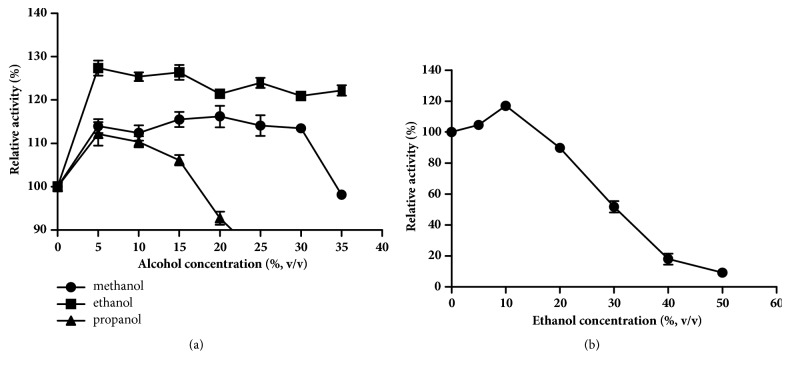
Effect of short-chain alcohols on the Bgl3B activity (a) and stability (b). Each value in the panel represents the mean ± SD (n = 3).

The effects of short-chain alcohols on the Bgl3B activity are shown Figure 3. The presence of alcohols can enhance the enzymatic activities distinctly; ethanol even enhanced the activities up to 130%. The optimal concentrations of methanol, ethanol, and propanol were found to be 20%, 15%, and 5% (v/v), respectively. When supplemented with different concentrations of ethanol, the Bgl3B retained stable in the presence of 0–25% ethanol, retaining more than 60% of the hydrolytic activity after incubation at 30°C for 4 h.

### 3.4. Substrate Specificity

The substrate specificities of the purified recombinant Bgl3B towards various substrates are shown in Table 2. The enzyme had significant activities of *β*-glucosidase, glucanase, cellobiase, xylanase, and isoflavone glycoside hydrolase. Of the tested substrates,* p*NPG was the most favorable substrate for Bgl3B, with the specific activities of 222.8 ± 6.7 U/mg. Besides the *β*-linked synthetic substrate* p*NPG, it was active towards natural substrate genistin and daidzin. Of the tested saccharides, cellobiose (*β*-1,4-linked) was the most suitable substrate, followed by laminaritetraose ([*β*-d-Glc-1,3)]_3_-d-linked), laminarin (*β*-1,3-linked), and lichenan (1,3:(1,4)_2_-*β*-d-linked).

### 3.5. Kinetic Parameters and Glucose Tolerance

When using* p*NPG and cellobiose as the substrates, the *K*_m_, *V*_max_, and *k*_cat_ values of Bgl3B were determined to be 1.03 and 7.63 mM, 469 and 526 *μ*mol/min/mg, and 714 and 800 /s, respectively. Catalytic efficiencies (*k*_cat_/*K*_m_) of Bgl3B against* p*NPG and cellobiose were 693 and 105 /mM/s, respectively. With* p*NPG as the substrate, Bgl3B showed a low* K*_*i*_ value (glucose inhibition constant), which was measured to be 7.1 mM.

### 3.6. Hydrolysis of Soybean Isoflavone Glycosides

Some *β*-glucosidases have capability of degrading soybean isoflavone glycosides [15]. HPLC analysis indicated that the soybean flour extract contained 133.0 mg/ml of isoflavone glucosides (daidzin, genistin, and glycitin) and 43.5 mg/ml of isoflavone aglycones (daidzein, genistein, and glycitein) (Table 3). When treating the soybean flour extract with Bgl3B at 37°C for 10 min, the amounts of the isoflavone glucosides were reduced to 23.3 mg/ml, while the amounts of isoflavone aglycones were increased to 72.4 mg/ml. Under the same conditions, the commercial *β*-glucosidase G4511 converted the same soybean isoflavones into 38.2 mg/ml isoflavone glycosides and 60.2 mg/ml free aglycones. It indicated that Bgl3B had greater soybean isoflavone glycosides-degrading ability than the commercial *β*-glucosidase from almond.

## 4. Discussion

The genus* Talaromyces* that is widely used in the production of lignocellulose-degrading enzymes with industrial purposes has a very promising prospect, and some* Talaromyces* species have been reported to be used for the production of thermotolerant *β*-glucosidases [7, 9, 23]. In our previous study, an acidic thermophilic GH3 *β*-glucosidase (Bgl3A, 737 aa) was identified in* T. leycettanus* JCM12802 [7]. In the present study, another GH3 *β*-glucosidase (Bgl3B, 860 aa) was also identified. Deduced Bgl3B and Bgl3A shared 75% sequence identity to each other but varied in enzymatic properties. Similar to most fungal *β*-glucosidases, recombinant Bgl3A and Bgl3B had acidic pH optima (4.5). However, Bgl3B remained highly stable over a wider pH range (pH 3.0–9.0 versus pH 4.0 of Bgl3A). Based on the multiple sequence alignments, recombinant Bgl3B had 24 putative* O*-glycosylation sites but lacked the key* O*-glycosylation sites (T436/T437/T443) of Bgl3A that is related to pH stability. The optimal temperature of Bgl3B (65°C) was the same as the *β*-glucosidase from* T. thermophiles *[9], but lower than Bgl3A (75°C) and Cel3a (71.5°C) from* T. emersonii *[23]. What is more, Bgl3B had better thermal stability than most *β*-glucosidases from thermophilic* T. piceus *[9],* Myceliophthora thermophile *[24], and* Humicola insolens *[25]. In addition, Bgl3B was highly resistant to various metal ions and reducing agents but showed susceptibility to Fe^3+^ and Ag^+^. These metal ions might affect the proper folding of Bgl3B, which has free -SH groups at the active sites [9].

The effect of short-chain alcohol on enzyme activity has been observed in *β*-glucosidases from* M. thermophile *[21],* Thermoascus aurantiacus *[26],* A. oryzae *[27], and* Fusarium oxysporum *[28]. Bgl3B showed similar enhanced activities in the presence of alcohols, with 15% ethanol as the best enhancer, and remained stable with ethanol of up to 10% (v/v). During the industrial ethanol production process, cellulases and ethanol coexist; a *β*-glucosidase with great ethanol tolerance is much favorable [29]. Therefore, the Bgl3B remaining highly active and stable in the presence of ethanol shows great application potentials in the bioethanol and wine industries.

According to the substrate specificity, *β*-glucosidases are classified into three major classes, i.e., aryl *β*-glucosidases, true cellobiases, and *β*-glucosidases with broad substrate specificity [30]. Bgl3B had high activities against both aryl *β*-glucosides and cellobiose, thus belonging to the third class. The catalytic efficiency (*k*_cat_/*K*_m_) of Bgl3B was 693 /mM/s towards* p*PNG, higher than that of *β*-glucosidases from* Aspergillus fumigatus *Z5 (125 /mM/s) [31],* Trichoderma koningiopsis *FCD3-1 (431 /mM/s) [6], and* Mucor circinelloides *NBRC4572 (286 /mM/s) [32]. For cellobiose, the *k*_cat_/*K*_m_ of Bgl3B was 104 /mM/s, higher than that of Bgl3A (75.8 /mM/s) from the same fungus and *β*-glucosidases from* A. fumigatus *Z5 (45 /mM/s) [31] and* M. circinelloides *NBRC4572 (54 /mM/s) [32]. Moreover, Bgl3B showed significant hydrolytic activities on various aryl *β*-glucosides and oligo- and polysaccharides as well as natural glucan substrates (laminarin and lichenan) and was weakly active on Avicel, CMC, and xylan. The results indicated that Bgl3B had capability of breaking the *β*-1,4 or *β*-1,3-glycosidic bonds. This broad substrate specificity makes Bgl3B suitable for use in diverse saccharide degradation processes [9].

Genistin and glycitin are the main isoflavones in soybean flour. The enzymatic conversion of soybean isoflavone glycosides into their aglycone forms using Bgl3B has been proved to be highly effective. After 10 min incubation at 37°C, 0.05 U of Bgl3B remarkably decreased the contents of daidzin, genistin, and glycitin with the conversion efficiency of daidzin (95.1%) > genistin (76.0%) > glycitin (75.3%). Under the same conditions, a commercial *β*-glucosidase from almonds showed less efficiency in isoflavone glycoside hydrolysis. Thus Bgl3B having high soy isoflavones-degrading capability is more promising for application in the different industries.

In summary, a novel GH3 *β*-glucosidase from thermophilic* T. leycettanus* was identified, heterologously produced, and biochemically characterized. The recombinant enzyme had acidic and thermostable properties and showed broader substrate specificity and higher catalytic efficiency on cellobiose than other known fungal counterparts. Moreover, it was highly efficient to convert isoflavone glycosides of soybean flour into their aglycone forms. These properties indicated that Bgl3B might be a promising enzyme in the food, feed, pharmacy, and biofuel industries, especially for the application to improve the nutritional value of soy products.

## Figures and Tables

**Table 1:** Effects of metal ions and chemical reagents (5 mM) on the activity of purified recombinant Bgl3B^a^.

Chemical	Relative activity (%)	Chemical	Relative activity (%)
Control	100.0 ± 1.5	Zn^2+^	94.2 ± 1.5
K^+^	100.1 ± 0.6	Mn^2+^	89.3 ± 1.9
Mg^2+^	98.3 ± 0.9	Cu^2+^	83.1 ± 0.3
Pb^2+^	98.1 ± 0.4	Fe^3+^	5.2 ± 0.3
Ni^2+^	97.8 ± 0.0	Ag^+^	ND
Na^+^	97.3 ± 0.3	*β*-Mercaptoethanol	95.6 ± 0.1
Ca^2+^	96.8 ± 1.1	EDTA	89.8 ± 1.1
Cr^3+^	96.2 ± 0.6	SDS	83.9 ± 0.3
Co^2+^	94.9 ± 0.2		

^a^The data are shown as the mean ± SD (n = 3); ND, not detected.

**Table 2:** The substrate specificity of the purified recombinant Bgl3B^a^.

Substrate	Glycosyl linkage	Specific activity (U/mg)	Relative activity (%)^a^
**Aryl-glycosides**			
*p*NPG (2 mM)	*β*-Glucose	222.8 ± 6.7	100.0
Genistin (1%)	*β*-Glucose	69.7 ± 0.2	31.3
Daidzin (1%)	*β*-Glucose	50.9 ± 0.2	22.8
Amygdalin (1%)	-	146.9 ± 0.1	65.9
**Oligosaccharides**			
Cellobiose (4 mM)	*β*-1,4-Glucose	189.5 ± 1.8	100.0
Cellotriose (1%)	*β*-1,4-Glucose	185.0 ± 4.1	97.6
Cellotetraose (1%)	*β*-1,4-Glucose	94.1 ± 2.1	49.7
Cellopentaose (1%)	*β*-1,4-Glucose	85.8 ± 1.5	45.3
Laminaritetraose (5 mM)	[*β*-d-Glc-1,3)]_3_-d-Glc	173.0 ± 4.1	91.3
**Polysaccharides**			
Laminarin (1%)	*β*-1,3-Glucan	25.7 ± 0.9	100.0
Lichenan (0.5%)	1,3:(1,4)_2_-*β*-d-Glucan	25.6 ± 0.6	99.8
Barley *β*-d-glucan (1%)	1,3:1,4-*β*-d-Glucan	7.2 ± 0.2	28.1
Avicel (1%)	*β*-1,4-Glucose	6.6 ± 0.2	25.5
Xylan (1%)	*β*-1,4-Xylose	4.1 ± 0.5	16.1
CMC-Na (1%)	*β*-1,4-Glucose	5.9 ± 0.4	23.0

^a^The data are shown as mean ± SD (n = 3). The specific activities of Bgl3B towards *p*NPG, cellobiose, and laminarin are defined as 100% for the aryl-glycosides, oligosaccharides, and polysaccharides, respectively.

**Table 3:** Conversion of soybean isoflavone glycosides into free isoflavones by Bgl3B and the commercial glucosidase (G4511) from Sigma-Aldrich^a^.

Enzyme	Isoflavone glycosides (mg/ml)			Free isoflavones (mg/ml)		
	Daidzin	Genistin	Glycitin	Daidzein	Genistein	Glycitein
Control	45.2 ± 1.9	80.5 ± 4.7	7.3 ± 1.2	26.4 ± 2.8	15.3 ± 1.6	1.8 ± 1.1
Bgl3B	2.2 ± 0.8	19.3 ± 4.2	1.8 ± 3.2	42.2 ± 2.7	26.5 ± 1.8	3.7 ± 0.4
G4511	6.7 ± 0.3	28.8 ± 2.7	2.7 ± 0.8	36.8 ± 1.5	20.4 ± 0.7	3.0 ± 0.7

^a^Results are the mean of three replicates. The reaction systems without enzyme addition were treated as blank controls.

## Supplementary Materials

Supplementary Materials Supplemental Table S1: primers used in this study. Supplemental Figure S1: multiple sequence alignment of Bgl3B with the GH3 *β*-glucosidases from* Thermoascus aurantiacus* (BGLI),* A. fumigatus* (5FJI), and* T. leycettanus* JCM12802 (Bgl3A). Identical and similar amino acids are indicated by black and gray boxes, respectively. The catalytic residues, nucleophile Asp261 and acid/base residue Glu490, are indicated. Supplemental Figure S2: the homology modeled structure of Bgl3B with the crystal structure of *β*-glucosidase from* A. fumigatus *(5FJI) as the template. The N-terminal catalytic domain (TIM-barrel), the C-terminal catalytic domain (*α*/*β* sandwich), and the fibronectin type III domain (Fn3) are indicated in green, yellow, and blue, respectively. Supplemental Figure S3: HPLC chromatograms of the soybean isoflavones. The purple line indicates the soybean flour extract; the red line indicates the soybean flour extract hydrolyzed by 0.05 U of Bgl3B at 37°C for 10 min; the black line indicates the soybean flour extract hydrolyzed by 0.05 U of commercial *β*-glucosidase G4511 from Sigma-Aldrich at 37°C for 10 min.
