# Supplementary material for: A Novel Thermostable GH3 β-Glucosidase from Talaromyce leycettanus with Broad Substrate Specificity and Significant Soybean Isoflavone Glycosides-Hydrolyzing Capability
Source: Biomed Res Int. 2018 Oct 23;2018:4794690. doi: 10.1155/2018/4794690 (PMC6218797; doi:10.1155/2018/4794690)

Supplementary Materials

A novel thermostable GH 3 β-glucosidase from *Talaromyce leycettanus* with broad substrate specificity and significant of soybean isoflavone glycosides-hydrolyzing capability

Xinxin Li[1 §], Wei Xia[2 §], Yingguo Bai[1], Rui Ma[1], Hong Yang[1], Huiying Luo[1], Pengjun Shi[1*], Bin Yao[1*]

[1]*Key Laboratory for Feed Biotechnology of the Ministry of Agriculture, Feed Research Institute, Chinese Academy of Agricultural Sciences, Beijing 100081, P. R. China*

[2] *State Key Laboratory of Food Science and Technology, Jiangnan University, Wuxi 214122, P. R. China*

[§]X. Li and W.Xia contributed equally to this paper.

[*]Corresponding authors. Address: Key Laboratory for Feed Biotechnology of the Ministry of Agriculture, Feed Research Institute, Chinese Academy of Agricultural Sciences, No. 12 Zhongguancun South Street, Beijing 100081, P. R. China. Tel: +86 10 82106053; fax: +86 10 82106054. E-mail addresses: shipengjun@caas.cn, binyao@caas.cn.

Table S1: Primers used in this study.

Reactions	Names	Sequences(5′→3′) [a]
The core region PCR	DP-F	GGCCGCAAYTGGGARGGNTT
	DP-R	GTCACCAGGCATNGHCATRTC
The upstream TAIL-PCR	SF1	TGAAGGGTCTTGTCGTCAATGTTGGAGCTAAT
	SF2	GGCAATAACACCGACATCCTGAATACCCTTG
The downstream TAIL-PCR	SR1	TCACGAATTGTACCTGTGGCCCTTTGCGGAT
	SR2	GCTACACGATGAACAAGCTGCTCAAAGGTGA

[a] The letters R, Y, N, and H represent A/G, C/T, A/T/C/G, and A/T/C, respectively.

Figure S1: Multiple sequence alignment of Bgl3B with BGLI, 5FJI and Bgl3A. Identical and similar amino acids are indicated by black and gray boxes, respectively. The catalytic residues, nucleophile Asp261 and acid/base Glu490, are also indicated.

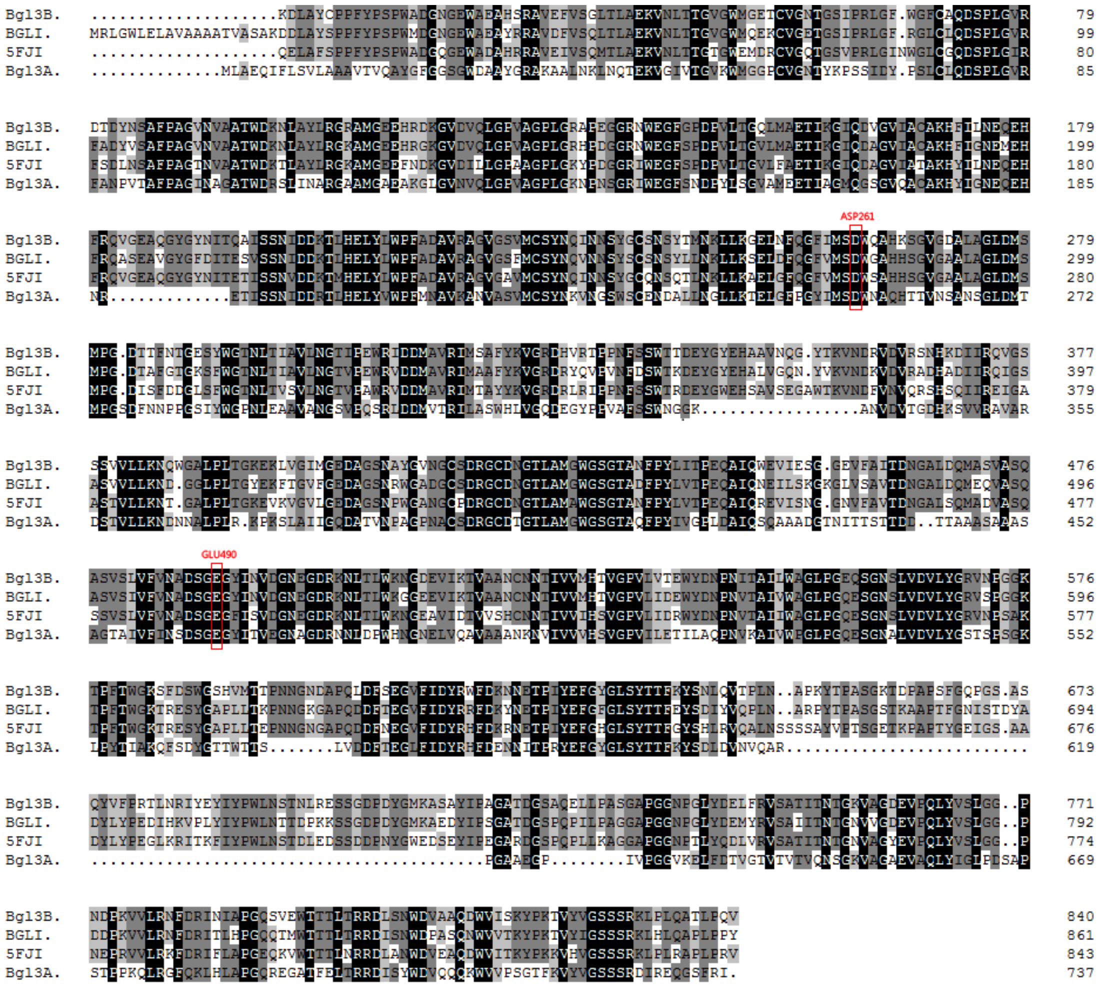

Figure S2: The homology modeled structure of Bgl3B with the crystal structure of β-glucosidase from A. *fumigatus* (5FJI) as the template. The N-terminal catalytic domain (TIM-barrel), the C-terminal catalytic domain (α/β sandwich), and the fibronectin type III domain (Fn3) are indicated in green, yellow and blue, respectively.

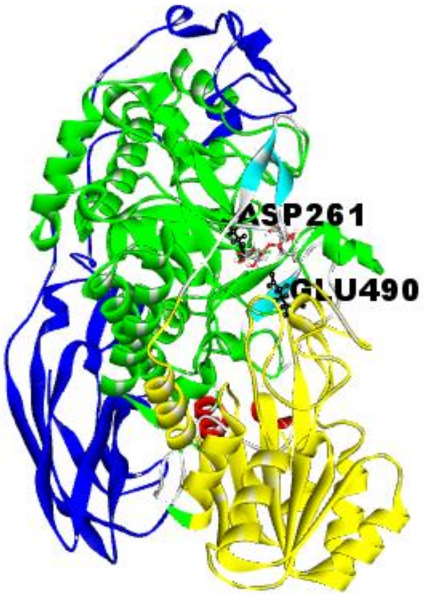

Figure S3: HPLC chromatograms of isoflavones. The purple line indicates the isoflavones in soybean flour extract; the red line indicates soybean flour extract hydrolyzed with 0.05 U of Bgl3B at 37 ℃ for 10 min; the black line indicates soybean flour extract hydrolyzed with 0.05 U of commercial β-glucosidase G4511 at 37 ℃ for 10 min.

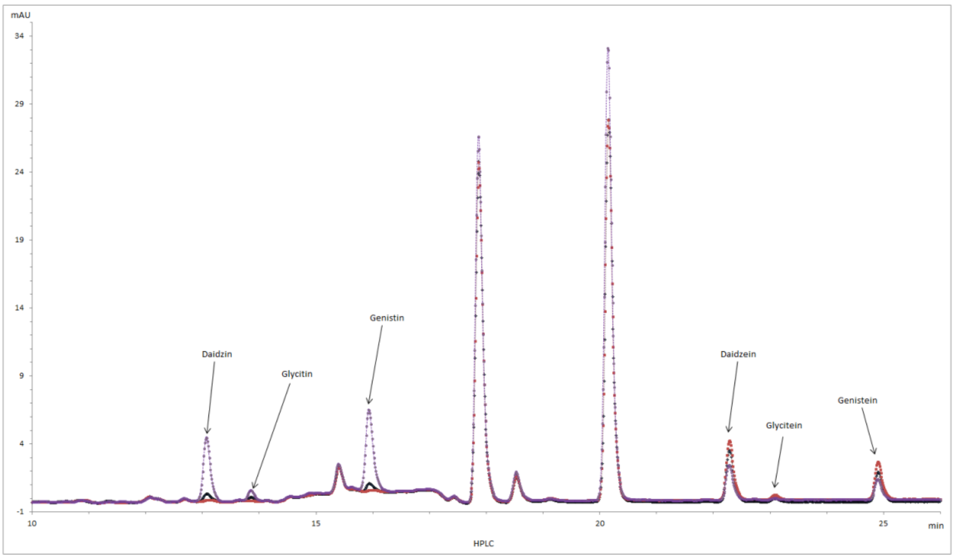

Supplement: Supplementary Materials — Supplemental Table S1: primers used in this study. Supplemental Figure S1: multiple sequence alignment of Bgl3B with the GH3 β-glucosidases from Thermoascus aurantiacus (BGLI), A. fumigatus (5FJI), and T. leycettanus JCM12802 (Bgl3A). Identical and similar amino acids are indicated by black and gray boxes, respectively. The catalytic residues, nucleophile Asp261 and acid/base residue Glu490, are indicated. Supplemental Figure S2: the homology modeled structure of Bgl3B with the crystal structure of β-glucosidase from A. fumigatus (5FJI) as the template. The N-terminal catalytic domain (TIM-barrel), the C-terminal catalytic domain (α/β sandwich), and the fibronectin type III domain (Fn3) are indicated in green, yellow, and blue, respectively. Supplemental Figure S3: HPLC chromatograms of the soybean isoflavones. The purple line indicates the soybean flour extract; the red line indicates the soybean flour extract hydrolyzed by 0.05 U of Bgl3B at 37°C for 10 min; the black line indicates the soybean flour extract hydrolyzed by 0.05 U of commercial β-glucosidase G4511 from Sigma-Aldrich at 37°C for 10 min. [file 4794690.f1.pdf]
